# Synthesis, Biological Evaluation and Docking Analysis of Some Novel Quinazolin Derivatives as Antitumor Agents

**Published:** 2016

**Authors:** Walaa S. El-serwy, Neama A. Mohamed, Emad M. M. Kassem, Khaled Mahmoud, M. M Mounier

**Affiliations:** a *Therapeutical Chemistry Department, National Research Center, Dokki, Cairo, Egypt.*; b *Department of Pharmacognosy, National Research Center, Cairo, Egypt.*

**Keywords:** Cytotoxic activity, Benzoxazin, Quinazolin, Antitumor, Docking analysis

## Abstract

Different acid chlorides (2a-d) reacted with anthranilic acid to produce 2-substituted-3, 1-benzoxazin-4-one (3a-d) which was used as starting material to synthesize some condensed and non-condensed heterocyclic compounds by reaction with nitrogen nucleophiles *e.g.,* hydrazine hydrate, and formamide. Some of the newly synthesized analogues were chosen to evaluate their cytotoxic activity against human carcinoma cell lines (HePG2– MCF7– A549). The docking and the cytotoxic activity results revealed that nearly all of the compounds containing *N*-phenyl aniline showed signiﬁcant inhibition for the three cell lines.

## Introduction

The synthesis of quinazolinone heterocycles has become the cornerstone for synthetic chemists and gained extensive importance in medicinal chemistry because of their diverse pharmacological activities including anti-mycobacterial (1-3), anti-fungal (4), antimalarial (5), antihypertensive (6-8), anti-histaminic (9-13), cardiotonic (14), anticancer (15-17), antiviral (18) and thymidylate synthase inhibitory activities (19, 20).

Substituted quinazolin-3(4*H*)-ones are among the versatile heterocyclic compounds, as they have a broad spectrum of pharmacological activities like anti-inflammatory (21), anticonvulsant (22-24), analgesic (25), antitubercular (26, 27) and anticancer activities (28-32). 

Benzoxazine heterocyclic compounds are potent non-steroidal progesterone receptor agonists (33) having many other activities such as anticancer, antiangiogenic (34), antidiabetic and hypolipidemic (35), antidepressant (36) and antiplatelet aggregation activities (37). 

Epidermal growth factor receptor (EGFR), which is cellular trans-membrane tyrosine kinase, is over-expressed in a signiﬁcant number of human tumors (*e.g*., breast, ovarian, colon, and prostate). An EGFR expression level often correlates with vascularity, and is associated with poor prognosis in patients. Inhibitors of the EGFR protein tyrosine kinase are, therefore, expected to have great therapeutic potential in the treatment of malignant and nonmalignant epithelial diseases (38-43). These ﬁndings encourage us to synthesize novel 3, 1-benzoxazin-4-one derivatives.

## Experimental

*Chemistry*

 All melting points are uncorrected and were taken on electro-thermal capillary melting point apparatus. Infrared spectra were recorded on a Jasco FT/IR-6100, Fourier transforms, Infrared spectrometer (Japan) at cm^-1^ scale using the KBr disc technique in the Central Services Laboratory, National Research Center, Dokki, Cairo, Egypt. ^1^H NMR spectra were determined by using a JEOl EX-270 NMR spectrometer (Japan) at the Central Services Laboratory, National Research Center, Dokki, Cairo, Egypt. The mass spectra were measured with a Finnigan MAT SSQ-7000 mass spectrometer at the Central Services Laboratory, Cairo University, Giza, Egypt. Follow up of the reactions and checking the purity of the compounds were made by TLC on silica gel-precoated aluminum sheets (Type 60, F 254, Merck, Darmstadt, Germany) and the spots were detected by exposure to UV analysis lamp at λ 254/366 nm for few seconds.

*General procedure for the preparation of compounds (3a, c, d)*

A solution of acid chloride (2a, c, d) (0.01 mol) and anthranilic acid (0.01 mol) in dry pyridine (30 mL) was refluxed for 3 h, the reaction mixture was cooled and poured into cold diluted HCl. The precipitate was collected by filtration and recrystallized from a proper solvent to give (3a, c, d). Spectroscopic data for all the compounds are given below.

*2-(Pyridin-3-yl)-4H-3, 1-benzoxazin-4-one* *(3a)*

Yield 80%. Yellow, white crystals. mp. 210-217 ˚C, IR (KBr, cm^-1^): 1700 (C = O). ^1^H NMR (DMSO-d_6_, δ ppm): 7.50-9.03 (m, 8H, aromatic). MS: (m/z) ≈ 224 (10%). Anal. Calcd for C_13_H_8_N_2_O_2_ (224.21): C, 69.64; H, 3.60; N, 12.49%. Found: C, 69.43; H, 3.44; N, 12.14%.

*2-(Pyridin-4-yl)-4H-3, 1-benzoxazin-4-one* *(3c)*

Yield 85%. Yellow crystals. mp >300 ˚C, IR (KBr, cm^-1^): 1692 (C = O). ^1^H NMR (DMSO-d_6_, δ ppm): 7.42-9.21 (m, 8H, aromatic). MS: (m/z) ≈ 224 (15%). Anal. Calcd for C_13_H_8_N_2_O_2_ (224.21): C, 69.64; H, 3.60; N, 12.49%. Found: C, 69.55; H, 3.51; N, 12.25%.

*2-[2-(Phenylamino) phenyl]-4H-3, 1-benzoxazin-4-one* *(3d)*

Yield 85%. Yellow crystals. mp. 235-240 ˚C, IR (KBr, cm^-1^): 1690 (C = O) and 3170 (NH). ^1^H NMR (DMSO-d_6_, δ ppm): 7.20-8.20 (m, 13H, aromatic), 11.72 (s, 1H, NH, exchangeable with D_2_O). MS: (m/z) ≈ 314 (5%). Anal. Calcd for C_20_H_14_N_2_O_2_ (314.33): C, 76.42; H, 4.49; N, 8.91%. Found: C, 76.03; H, 4.20; N, 8.34%.

*General procedure for the preparation of compounds (4a, b)*

A mixture of (3a (44), 3b (45)) (0.01 mol) and formamide (0.015 mol) was refluxed for 3 h in boiling ethanol (30 mL), then poured into water. The precipitated solid after concentration and cooling was collected by filtration and crystallized from the proper solvent to give (4a, b). Spectroscopic data for all the compounds are given below.

*2-(Pyridin-3-yl) quinazolin-4 (3H)-one* *(4a)* *:*

Yield 65%, White crystals. mp. >300 ˚C, IR (KBr, cm^-1^): 1700 (C = O) and 3299 (NH). ^1^H NMR (DMSO-d_6_, δ ppm): 7.23-8.32 (m, 8H, aromatic), 12 (s, 1H, NH, exchangeable with D_2_O). MS: (m/z) ≈ 223 (0.13%). Anal. Calcd for C_13_H_9_N_3_O (223.23): C, 69.95; H, 4.06; N, 18.82%. Found: C, 69.62; H, 3.88; N, 18.60%.

2-[(*E)-2-(furan-2-yl) ethenyl] quinazolin-4 (3H)-one (4b)*

Yield 85%. Black crystals. mp. 170-175 ˚C, IR (KBr, cm^-1^): 1698 (C = O) and 3150 (NH). ^1^H NMR (DMSO-d_6_, δ ppm): 6.48 (d,* J *= 5.4 Hz, 1H, CH), 6.89 (d,* J *= 2.7 Hz, 1H, CH), 7.11-8.59 (m, 7H, aromatic), 11.78 (s, 1H, NH, exchangeable with D_2_O).MS: (m/z) ≈ 238 (10%). Anal. Calcd for C_14_H_10_N_2_O_2_ (238.24): C, 70.58; H, 4.23; N, 11.76%. Found: C, 70.30; H, 4.08; N, 11.50%.

*General procedure for the preparation of compounds (5a, b)*

A mixture of (4a, b) (0.01 mol) and chloroacetyl chloride (0.01 mol) was refluxed in boiling *N, N*-dimethylformamide (DMF) (30 mL) for 3 h. Then the mixture was poured into water. The precipitate was collected by filtration, dried and crystallized from the proper solvent to give (5a, b). Spectroscopic data for all the compounds are given below.

*3-(Chloroacetyl)-2-(pyridin-3-yl) quinazolin-4 (3H)-one* *(5a)*

Yield 80%. Gray crystals. mp. >300 ˚C, IR (KBr, cm^-1^): 1650 (C = O) and 1690 (C = O). ^1^H NMR (DMSO-d_6_, δ ppm): 4.48 (s, 2H, CH_2_), 7.63-9.07 (m, 8H, aromatic). MS: (m/z) ≈ 299 (6%), [M + 2]^+^ m/z ≈ 301 (3%). Anal. Calcd for C_15_H_10_ClN_3_O_2_ (299.71): C, 60.11; H, 3.36; N, 14.02%. Found: C, 59.90; H, 2.98; N, 13.90%.

*3-(Chloroacetyl)-2-[(E)-2-(furan-2-yl) ethenyl] quinazolin-4 (3H)-one* *(5b)*

Yield 90%. Black crystals. mp. 151-155 ˚C, IR (KBr, cm^-1^): 1690 (C = O) and 1710 (C = O). ^1^H NMR (DMSO-d_6_, δ ppm): 4.90 (s, 2H, CH_2_), 6.23 (d,* J *= 8.1 Hz, 1H, CH), 6.70 (d,* J *= 5.4 Hz, 1H, CH), 6.95-8.21 (m, 7H, aromatic). MS: (m/z) ≈ 314 (1.8%), [M+2] ^+^ m/z ≈ 316 (1%). Anal. Calcd for C_16_H_11_ClN_2_O_3_ (314.72): C, 61.06; H, 3.52; N, 8.90%. Found: C, 60.90; H, 3.30; N, 8.67%.

*General procedure for the preparation of compounds (6a, b)*

A mixture of (5a, b) (0.01 mol) and hydrazine hydrate (0.015 mol) was heated in boiling ethanol (30 mL) under reflux for 4 h. Then the mixture was poured into water. The precipitate was collected by filtration, dried and crystallized from the proper solvent to give (6a, b). Spectroscopic data for all the compounds are given below. 

*3-(Hydrazinylacetyl)-2-(pyridin-3-yl) quinazolin-4 (3H)-one* *(6a)*

Yield 75%. Gray crystals. mp. 106-110 ˚C, IR (KBr, cm^-1^): 1690, 1700 (2C = O), 3190 (NH) and 3300-3444 (NH_2_). ^1^H NMR (DMSO-d_6_, δ ppm): 3.55 (s, 2H, CH_2_), 3.80 (s, 2H, NH_2_, exchangeable with D_2_O), 7.58-9.07 (m, 8H, aromatic), 10.49 (s, 1H, NH, exchangeable with D_2_O). MS: (m/z) ≈ 295 (12%). Anal. Calcd for C_15_H_13_N_5_O_2_ (295.29): C, 61.01; H, 4.44; N, 23.72%. Found: C, 60.85; H, 4.20; N, 23.50%.

*2-[(E)-2-(furan-2-yl) ethenyl]-3-(hydrazinylacetyl) quinazolin-4 (3H)-one* *(6b)*

Yield 65%. White crystals. mp. > 300 ˚C, IR (KBr, cm^-1^): 1687, 1697 (2C = O), 3174 (NH) and 3320-3400 (NH_2_). ^1^H NMR (DMSO-d_6_, δ ppm): 3.49 (s, 2H, CH_2_), 3.70 (s, 2H, NH_2_, exchangeable with D_2_O), 6.65, 6.90 (2d,* J *= 5.4 Hz,* J *= 2.7 Hz, 2H, 2CH), 7.01-8.48 (m, 7H, aromatic), 11.21 (s, 1H, NH, exchangeable with D_2_O). MS: (m/z) ≈ 310 (3%). Anal. Calcd for C_16_H_14_N_4_O_3 _(310.30): C, 61.93; H, 4.55; N, 18.06%. Found: C, 61.70; H, 4.35; N, 17.80%.

*General procedure for the preparation of compounds (7c, d)*

A solution of (3c, d) (44) (0.01 mol) in dry benzene (30 mL) and hydrazine hydrate (0.015 mol) was heated under reflux for 4 h. Then the mixture was poured into water. The precipitate was collected by filtration, dried and crystallized from the proper solvent to give (7c, d) (44). Spectroscopic data for all the compounds are given below.

*3-Amino-2-(pyridin-4-yl) quinazolin-4 (3H)-one* *(7c)*

Yield 75%, Black crystals. mp. 150-155 ˚C, IR (KBr, cm^-1^): 1685 (C = O) and 3311-3420 (NH_2_). ^1^H NMR (DMSO-d_6_, δ ppm): 7.68-8.66 (m, 8H, aromatic), 10.08 (s, 2H, NH_2_, exchangeable with D_2_O). MS: (m/z) ≈ 238 (15%). Anal. Calcd for C_13_H_10_N_4_O (238.24): C, 65.54; H, 4.23; N, 23.52%. Found: C, 65.32; H, 4.18; N, 23.40%.

*3-Amino-2-[2-(phenylamino) phenyl] quinazolin-4 (3H)-one* *(7d)*

Yield 85%. Yellow crystals. mp. 260-265 ˚C, IR (KBr, cm^-1^): 1700 (C = O), 3172 (NH) and 3300-3434 (NH_2_). ^1^H NMR (DMSO-d_6_, δ ppm): 3.60 (s, 2H, NH_2_, exchangeable with D_2_O), 6.68-8.54 (m, 13H, aromatic), 12.01 (s, 1H, NH, exchangeable with D_2_O). MS: (m/z) ≈ 328 (20%). Anal. Calcd for C_20_H_16_N_4_O (328.36): C, 73.15; H, 4.91; N, 17.06%. Found: C, 73.01; H, 4.75; N, 16.90%.

*General procedure for the preparation of compounds (8c, d)*

A solution of (7c, d) (44) (0.01 mol), was allowed to react with chloroacetyl chloride (0.01 mol) in refluxing pyridine about 2 h, and then poured over ice/HCl. The precipitate was collected by filtration and crystallized from the proper solvent to give (8c, d). Spectroscopic data for all the compounds are given below.

*2-Chloro-N-[4-oxo-2-(pyridin-4-yl) quinazolin-3 (4H)-yl] acetamide* *(8c)*

Yield 70%. Yellow crystals. mp. > 300 ˚C, IR (KBr, cm^-1^): 1698, 1715 (2C = O) and 3175 (NH). ^1^H NMR (DMSO-d_6_, δ ppm): 4.78 (s, 2H, CH_2_), 7.65-8.44 (m, 8H, aromatic), 11.87 (s, 1H, NH, exchangeable with D_2_O). MS: (m/z) ≈ 314 (8%), [M + 2] ^+^ m/z ≈ 316 (4%). Anal. Calcd for C_15_H_11_ClN_4_O_2_ (314.72): C, 57.24; H, 3.52; N, 17.80%. Found: C, 57.12; H, 3.40; N, 17.60%.

*2-Chloro-N-{4-oxo-2-[2-(phenylamino) phenyl] quinazolin-3 (4H)-yl} acetamide* *(8d)*

Yield 75%. Black crystals. mp. 190-195 ˚C, IR (KBr, cm^-1^): 1677, 1690 (2C = O) and 3230 (NH). ^1^H NMR (DMSO-d_6_, δ ppm): 4.90 (s, 2H, CH_2_), 6.81-8.20 (m, 13H, aromatic), 11.90, 12 (2s, 2H, 2NH, exchangeable with D_2_O). MS: (m/z) ≈ 404 (23%), [M + 2] ^+^ m/z ≈ 406 (15%). Anal. Calcd for C_22_H_17_ClN_4_O_2_ (404.84): C, 65.27; H, 4.23; N, 13.84%. Found: C, 65.05; H, 4.18; N, 13.75%.

*General procedure for the preparation of compounds (9c, d)*

A solution of compounds (7c, d) (44) (0.01 mol) and chloroacetamide (0.015 mol) was refluxed for 3 h in boiling *N, N*-dimethylformamide (DMF) (30 mL). Then the mixture was poured into water. The precipitate was collected by filtration, dried and crystallized from the proper solvent to give (9c, d). Spectroscopic data for all the compounds are given below.

*6-(Pyridin-4-yl)-3, 4-dihydro-2H-[1, 2, 4] triazino [2, 3-c] quinazolin-2-one* *(9c)*

Yield 65%. Black crystals. mp. > 300 ˚C, IR (KBr, cm^-1^): 1710 (C = O) and 3189 (NH). ^1^H NMR (DMSO-d_6_, δ ppm): 3.76 (s, 2H, CH_2_), 7.33-8.66 (m, 8H, aromatic), 10.70 (s, 1H, NH, exchangeable with D_2_O). MS: (m/z) ≈ 277 (13%). Anal. Calcd for C_15_H_11_N_5_O (277.28): C, 64.97; H, 4.00; N, 25.26%. Found: C, 64.70; H, 3.88; N, 25.07%.

*6-[2-(Phenylamino) phenyl]-3, 4-dihydro-2H-[1, 2, 4] triazino [2, 3-c] quinazolin-2-one* *(9d)*

Yield 85%. Yellow crystals. mp. 256-260 ˚C, IR (KBr, cm^-1^): 1677 (C = O) and 3150 (NH). ^1^H NMR (DMSO-d_6_, δ ppm): 3.65 (s, 2H, CH_2_), 6.87-7.96 (m, 13H, aromatic), 10.70, 11.30 (2s, 2H, 2NH, exchangeable with D_2_O). MS: (m/z) ≈ 367 (19%). Anal. Calcd for C_22_H_17_N_5_O (367.40): C, 71.92; H, 4.66; N, 19.06%. Found: C, 71.76; H, 4.49; N, 18.89%.

*General procedure for the preparation of compounds (10c, d)*

A solution of compounds (7c, d) (44) (0.01 mol) and phenyl isothiocyanate (0.01 mol) was refluxed in boiling benzene (30 mL) for 3 h, then concentrated and crystallized from the proper solvent to give (10c, d). Spectroscopic data for all the compounds are given below.

*1-[4-Oxo-2-(pyridin-4-yl) quinazolin-3 (4H)-yl]-3-phenylthiourea* *(10c)*

Yield 90%. White crystals. mp. 195-200 ˚C, IR (KBr, cm^-1^): 1685 (C = O) and 3190 (NH). ^1^H NMR (DMSO-d_6_, δ ppm): 7.33-8.96 (m, 13H, aromatic), 10.49, 11.01 (2s, 2H, 2NH, exchangeable with D_2_O). MS: (m/z) ≈ 373 (5%). Anal. Calcd for C_20_H_15_N_5_OS (373.43): C, 64.33; H, 4.05; N, 18.75%. Found: C, 64.12; H, 3.90; N, 18.50%.

*1-(4-Oxo-2-(2-(phenylamino) phenyl) quinazolin-3 (4H)-yl)-3-phenylthiourea* *(10d)*

Yield 80%. Yellow crystals. mp. 200-205 ˚C, IR (KBr, cm^-1^): 1700 (C = O) and 3200 (NH). ^1^H NMR (DMSO-d_6_, δ ppm): 7.09-8.24 (m, 18H, aromatic), 9.77, 9.86, 11.70 (3s, 3H, 3NH, exchangeable with D_2_O). MS: (m/z) ≈ 463 (3%). Anal. Calcd for C_27_H_21_N_5_OS (463.55): C, 69.96; H, 4.57; N, 15.11%. Found: C, 69.69; H, 4.48; N, 14.90%.

*General procedure for the preparation of compounds (11c, d)*

A solution of (7c, d) (44) (0.01 mol) and benzoyl chloride (0.01 mol) in dry acetone (30 mL) was refluxed for 3 h. Excess solvent was removed and The precipitated solid obtained was crystallized from suitable solvent to obtain (11c, d). Spectroscopic data for all the compounds are given below.

*N-[4-oxo-2-(pyridin-4-yl) quinazolin-3 (4H)-yl] benzamide* *(11c)*

Yield 70%. Yellow crystals. mp. 180-185 ˚C, IR (KBr, cm^-1^): 1677, 1690 (2C = O) and 3150 (NH). ^1^H NMR (DMSO-d_6_, δ ppm): 7.31-8.42 (m, 13H, aromatic), 12.01 (s, 1H, NH, exchangeable with D_2_O). MS: (m/z) ≈ 342 (17%). Anal. Calcd for C_20_H_14_N_4_O_2_ (342.35): C, 70.17; H, 4.12; N, 16.37%. Found: C, 70.02; H, 3.90; N, 16.17%.

*N-(4-oxo-2-(2-(phenylamino) phenyl) quinazolin-3 (4H)-yl) benzamide* *(11d)*

Yield 80%. Yellow crystals. mp. > 300 ˚C, IR (KBr, cm^-1^): 1687, 1693 (2C = O) and 3177 (NH). ^1^H NMR (DMSO-d_6_, δ ppm): 7.16-8.45 (m, 18H, aromatic), 11.01, 12.01 (2s, 2H, 2NH, exchangeable with D_2_O). MS: (m/z) ≈ 432 (10%). Anal. Calcd for C_27_H_20_N_4_O_2_ (432.47): C, 74.98; H, 4.66; N, 12.95%. Found: C, 74.70; H, 4.50; N, 12.80%.

*General procedure for the preparation of compounds (12c, d)*

A solution of (11c, d) (0.01 mol) with ammonium acetate (0.01 mol) in acetic acid (30 mL) was heated under reflux for 3 h, then poured into water. The precipitated solid after concentration and cooling was collected by filtration and crystallized from suitable solvent to give (12c, d). Spectroscopic data for all the compounds are given below.

*2-Phenyl-5-(pyridin-4-yl) [1, 2, 4] triazolo [1, 5-c] quinazoline* *(12c)*

Yield 65%. Gray crystals. mp. 215-220 ˚C, ^1^H NMR (DMSO-d_6_, δ ppm): 7.41-8.75 (m, 13H, aromatic). MS: (m/z) ≈ 323 (33%). Anal. Calcd for C_20_H_13_N_5_ (323.35): C, 74.29; H, 4.05; N, 21.66%. Found: C, 74.11; H, 3.89; N, 21.56%.

*N-phenyl-2-(2-phenyl-[1, 2, 4] triazolo [1, 5-c] quinazolin-5-yl) aniline* *(12d)*

Yield 85%. Yellow crystals. mp. 240-245 ˚C, IR (KBr, cm^-1^): 3177 (NH). ^1^H NMR (DMSO-d_6_, δ ppm): 6.69-8.28 (m, 18H, aromatic), 13 (s, 1H, NH, exchangeable with D_2_O). MS: (m/z) ≈ 413 (11%). Anal. Calcd for C_27_H_19_N_5_ (413.47): C, 78.43; H, 4.63; N, 16.94%. Found: C, 78.22; H, 4.48; N, 16.80%.

*Cytotoxic effect on human cell line (HePG2 – MCF 7 - A549)*

Cell viability was assessed by the mitochondrial dependent reduction of yellow MTT (3-(4, 5-dimethylthiazol-2-yl)-2, 5-diphenyl tetrazolium bromide) to purple formazan (46). 

Procedure: All the following procedures were done in a sterile area using a Laminar flow cabinet biosafety class II level (Baker, SG403INT, Sanford, ME, USA). Cells were suspended in RPMI 1640 medium for HePG2- MCF7 and DMEM for A549. The media are supplemented with 1% antibiotic-antimycotic mixture (10,000 U/mL Potassium Penicillin, 10,000 µg/mL Streptomycin Sulfate and 25 µg/mL Amphotericin B), 1% L-glutamine and 10% fetal bovine serum and kept at 37 ºC under 5% CO_2_.

Cells were batch cultured for 10 days, then seeded at concentration of 10x10^3^ cells/well in fresh complete growth medium in 96-well Microtiter plastic plates at 37 ˚C for 24 h under 5% CO_2_ using a water jacketed Carbon dioxide incubator (Sheldon, TC2323, Cornelius, OR, USA). Media was aspirated, fresh medium (without serum) was added and cells were incubated either alone (negative control) or with different concentrations of sample to give a final concentration of (100-50-25-12.5-6.25-3.125-0.78 and 1.56 µg/mL). After 48 h of incubation, the medium was aspirated, 40 µL MTT salt (2.5 μg/mL) were added to each well and incubated for a further four hours at 37 ºC under 5% CO_2_. To stop the reaction and dissolving the formed crystals, 200 μL of 10% Sodium dodecyl sulphate (SDS) in deionized water was added to each well and incubated overnight at 37 ºC. A positive control which composed of 100 µg/mL was used as a known cytotoxic natural agent who gives 100% lethality under the same conditions (47, 48).

The absorbance was then measured using a microplate multi-well reader (Bio-Rad Laboratories Inc., model 3350, Hercules, California, USA) at 595 nm and a reference wavelength of 620 nm. A statistical significance was tested between samples and negative control (cells with vehicle) using independent t-test by SPSS 11 program. DMSO is the vehicle used for dissolution of plant extracts and its final concentration in the cells was less than 0.2%. The percentage of change in viability was calculated according to the formula: 

(Reading of extract/Reading of negative control)-1) x 100. A probit analysis was carried for IC_50_ and IC_90_ determination using SPSS 11 program.

*Molecular docking study*

All docking studies were performed using "Internal Coordinate Mechanics" (Molsoft ICM 3.5-0a). 

*Preparation of small molecule*

Compounds 2d, 3a, 3b, 3d, 4a, 4b, 5a, 5b, 6a, 6b, 7c, 7d, 8d, 9c, 9d, 10c, 10d, 11c, 11d, 12c, 12d were built in Chem Draw Ultra version 11.0 and their energy minimized through Chem3D Ultra version 11.0/MM2, Jop Type: minimum RMS Gradient of 0.100, and saved as MDL Mol File (^*^.Mol).

*Generation of Ligand and Enzyme Structures*

The crystal structures of EGFR (PDB code: 1M17) complex were retrieved from the RCSB Protein Data Bank (http://www.rcsb.org/pdb/ home/home.do). 

**Figure 1:**
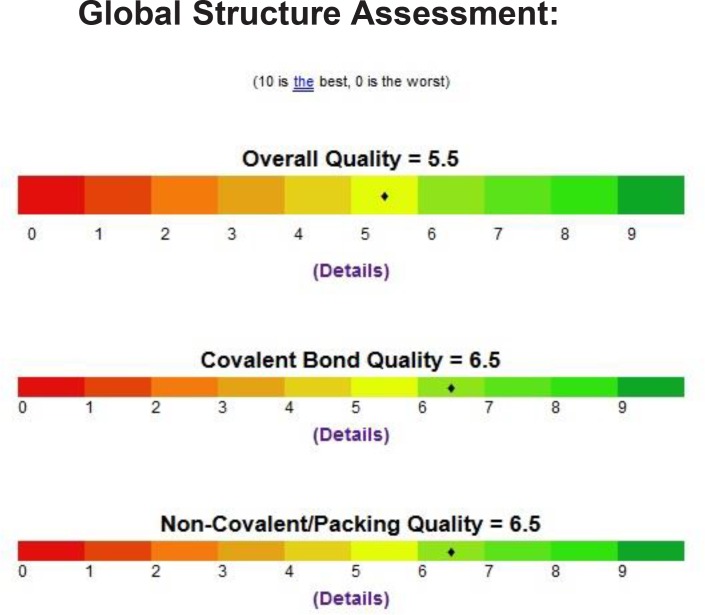
Quality of the PDB file that was used using the Prosess

**Figure 2:**
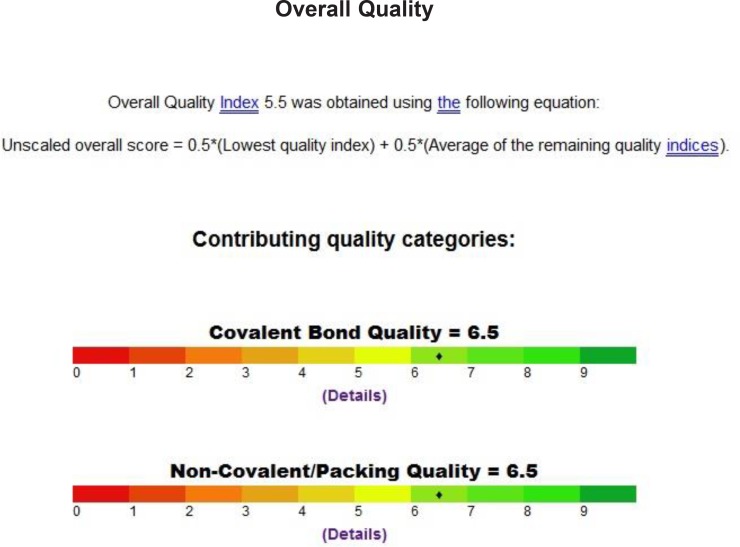
Quality of the PDB file that was used using the Prosess

**Figure 3:**
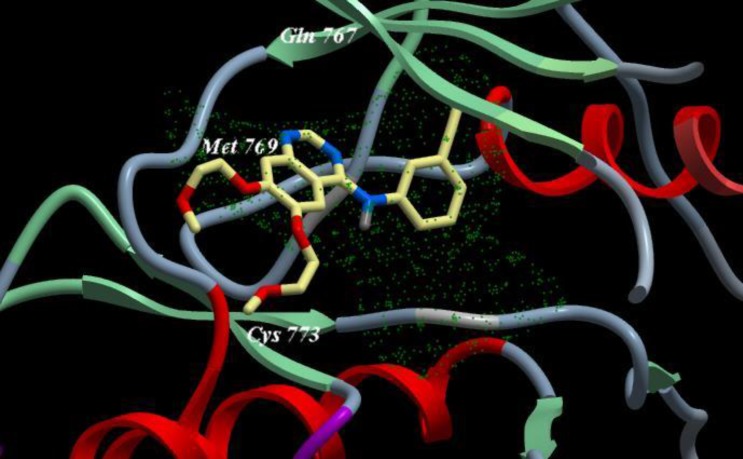
Binding model of erlotinib in to active pocket of EGFR receptor

We inspect the quality of the PDB file that was used using the PROSESS (Protein Structure Evaluation Suite & Server) (http://www.prosess.ca/) (Figure 1, 2). In our investigation, the 3D-coordinates in X-ray crystal structure of EGFR in complex with the ligand, Erlotinib (PDB entry 1M17) was used as the receptor model in EGFR docking simulation (Figure 3). All bound waters ligands and cofactors were removed from the protein. 

*Docking using Molsoft ICM 3.5-0 a program*

The conversion of our PDB file into an ICM object involves the addition of hydrogen bonds, assignment of atom types, and charges from the residue templates, then perform ICM small molecule docking through setup the receptor, review and adjust binding site makes receptor maps, then start docking simulation, followed by displaying the results. ICM stochastic global optimization algorithm attempts to find the global minimum of the energy function that include five grid potentials describing the interaction of the flexible ligand with the receptor and internal conformational energy of the ligand, during this process a stack of alternative low energy conformations is saved. All inhibitors were compared according to the best binding free energy (minimum) obtained among all the run. 

## Results and Discussion

*Chemistry*

**Scheme 1:**
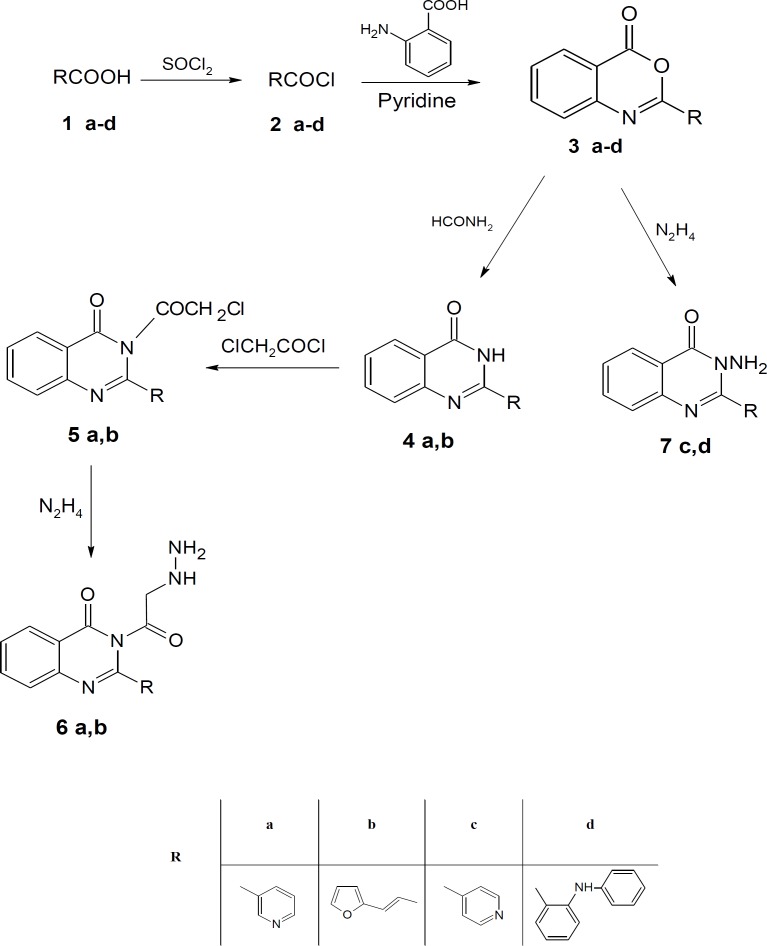
Synthesis of the newly 3, 1-benzoxazin-4-one derivatives

Different acid chlorides namely, pyridine-3-carbonyl chloride, (2*E*)-3-(furan-2-yl) prop-2-enoyl chloride, pyridine-4-carbonyl chloride and 2-(phenylamino) benzoyl chloride 2a-d, respectively reacted with anthranilic acid to produce 2-[substituted]-4*H*-3, 1-benzoxazin-4-one 3a-d (Scheme 1). Compounds 3a, b reacted with formamide to give 2-(substituted) quinazolin-4 (3*H*)-one 4a, b which reacted with chloroacetyl chloride to give 3-(chloroacetyl)-2-[substituted] quinazolin-4 (3*H*)-one 5a, b (Scheme 1). Compounds 5a, b reacted with hydrazine hydrate to give 3-(hydrazinylacetyl)-2-[substituted] quinazolin-4 (3*H*)-one 6a, b (Scheme 1). The structures of all of the newly synthesized derivatives were established via the elemental analyses and IR, ^1^H NMR and mass spectral data. IR spectra of the compounds 6a, b exhibited characteristic absorption bands in the range 3174-3444 cm^-1^ due to the respective NH and NH_2_. ^1^H NMR (DMSO-d_6_) spectra of compounds 6a, b revealed signals at δ 3.70-3.80 ppm and 10.49-11.21 ppm representing NH_2_ and NH groups, respectively.

**Scheme 2:**
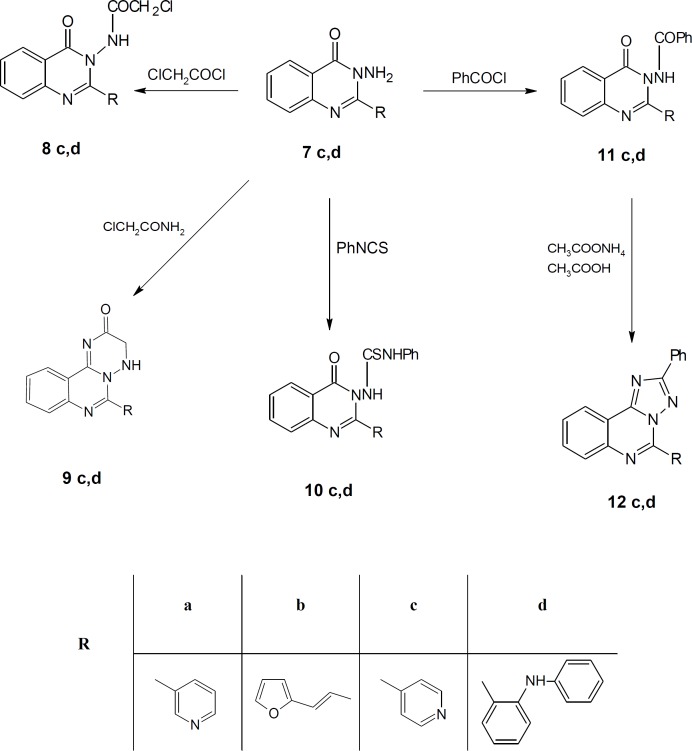
Synthesis of the newly 3, 1-benzoxazin-4-one derivatives

Also, compounds 3c, d reacted with hydrazine hydrate to give 3-amino-2-(substituted) quinazolin-4 (3*H*)-one 7c, d (Scheme 1) which reacted with chloroacetyl chloride to give 2-chloro-*N*-[4-oxo-2-(substituted) quinazolin-3 (4*H*)-yl] acetamide 8c, d (Scheme 2). IR spectra of the derivatives 8c, d exhibited the disappearance of the characteristic band of NH_2_ group and showed the presence of bands at the range 1690-1715 cm^-1^ corresponding to CO groups.

Finally, compounds 7c, d reacted with chloroacetamide, phenyl isothiocyanate and benzoyl chloride to give compounds 9-11 (c, d), respectively (Scheme 2). Compounds 11c, d reacted with ammonium acetate to give *N*-phenyl-2-(substituted-[1, 2, 4] triazolo [1, 5-c] quinazolin 12c, d (Scheme 2).

*In-vitro Antitumor Screening against A549, HePG2 and MCF7 cell lines*

The cytotoxic potencies of compounds 2d, 3a, 3b, 3d, 4a, 4b, 5a, 5b, 6a, 6b, 7c, 7d, 8d, 9c, 9d, 10c, 10d, 11c, 11d, 12c, 12d against a panel of three human tumor cell lines were investigated and compared with the reference drug doxorubicin (Table 1). The human tumor cell line panel consisted of breast carcinoma (MCF7), liver carcinoma (HePG2) and lung carcinoma (A549) using MTT assay. Tumor cells were incubated either alone (negative control) or with different concentrations of the test compounds (100–50–25–12.5–6.25–3.125–0.78 and 1.56 µM). With regard to sensitivity against individual cell lines, this class is more effective on hepatocellular carcinoma more than other two cell lines. Compound 10c showed selective potency against A549 cell line (IC_50 _= 72.2) as shown in Table 2. However, compounds 11d and 9d showed selective potency against HePG2 cell line with IC_50_ 53.4 and 66.7 µg/ml, respectively, as shown in Table 3 and compounds 4b and 8d for MCF7 cell line with IC_50 _81.9 and 90.5 µg/mL, respectively as shown in Table 4. However, compounds 7d, 3d and 2d showed effectiveness against all cell lines with IC_50 _(62.6, 85.0 and 92.1 µg/mL), (65.1, 82.9 and 77.6 µg/mL) and (75.8, 81.9 and 86.1 µg/mL) for HePG2, MCF7 and A549 as shown in Table 3, 4, 2, respectively. In addition, compounds 10d and 6a displayed selective potency against A549 and HePG2 cell lines with IC_50_ of 88.4, 92.1 and 45.6, 32.8 µg/mL concentrations, respectively as shown in Table 2, 3. While compound 12d displayed selective potency against HePG2 and MCF7 cell lines with IC_50_ 33.3 and 87.4 µg/mL, respectively. Moreover, Compounds 6a and 12d considered the most potent compounds against the HePG2 cell line, while compounds 7d, 3d, 9d, 10d, 11d and 2d possessed moderate antitumor activity compared to positive control doxorubicin. 

**Table 1:** Positive control Adrinamycin (Doxorubicin) [Mw = 579.99].

	**IC**_50_ ** (µg/mL)**
HEPG2	21.6
A549	28.3
MCF7	26.1
PC3	23.8

**Table 2:** Sample was tested against the human tumor cell line A549 [Lung carcinoma cell line].

**Remarks**	**IC**_90 _(µg/mL)	**IC**_50 _(µg/mL)	***Sample Code***
57.8% at 100ppm	137	86.1	*2d*
20.6% at 100ppm	--------	--------	*3a*
32.8% at 100ppm	--------	--------	*3b*
65.1% at 100ppm	126.3	77.6	*3d*
22.3% at 100ppm	--------	--------	*4a*
20.7% at 100ppm	--------	--------	*4b*
28.2% at 100ppm	--------	--------	*5a*
0% at 100ppm	--------	--------	*5b*
51.7% at 100ppm	145.4	92.1	*6a*
1.4% at 100ppm	--------	--------	*6b*
0% at 100ppm	--------	--------	*7c*
51.6% at 100ppm	144.2	92.1	*7d*
35.2% at 100ppm	--------	--------	*8d*
25.2% at 100ppm	--------	--------	*9c*
45.6% at 100ppm	--------	--------	*9d*
69.6% at 100ppm	121.4	72.2	*10c*
54.5% at 100ppm	142.2	88.4	*10d*
13.9% at 100ppm	--------	--------	*11c*
40.8% at 100ppm	--------	--------	*11d*
19.8% at 100ppm	--------	--------	*12c*
42.8% at 100ppm	--------	--------	*12d*
5% at 100ppm	--------	--------	*DMSO*
*0 %*	*--------*	*--------*	*Negative control*

IC_50_: Lethal concentration of the sample which causes the death of 50% of cells in 48 h.

IC_90_: Lethal concentration of the sample which causes the death of 90% of cells in 48 h.

**Table 3:** Sample was tested against the human tumor cell line HePG2 [Human hepatocellular carcinoma cell line

**Remarks**	**IC**_90 _(µg/mL)	**IC**_50 _(µg/mL)	***Sample Code***
70.5% at 100ppm	120.9	75.8	*2d*
-47% at 100ppm	--------	--------	*3a*
35.3% at 100ppm	--------	--------	*3b*
78.6% at 100ppm	109.6	65.1	*3d*
21.3% at 100ppm	--------	--------	*4a*
2.3% at 100ppm	--------	--------	*4b*
0% at 100ppm	--------	--------	*5a*
0% at 100ppm	--------	--------	*5b*
100% at 100ppm	57.1	32.8	*6a*
5.7% at 100ppm	--------	--------	*6b*
0% at 100ppm	--------	--------	*7c*
84.5% at 100ppm	103.9	62.6	*7d*
40.6% at 100ppm	--------	--------	*8d*
4.3% at 100ppm	--------	--------	*9c*
84.2% at 100ppm	104.8	66.7	*9d*
0% at 100ppm	--------	--------	*10c*
94.2% at 100ppm	79.1	45.6	*10d*
0% at 100ppm	--------	--------	*11c*
85.4% at 100ppm	95.2	53.4	*11d*
22.5% at 100ppm	--------	--------	*12c*
100% at 100ppm	59.9	33.3	*12d*
1% at 100ppm	--------	--------	*DMSO*
*0 %*	*--------*	*--------*	*Negative control*

IC_50_: Lethal concentration of the sample which causes the death of 50% of cells in 48 h.

IC_90_: Lethal concentration of the sample which causes the death of 90% of cells in 48 h.

**Table 4:** Sample was tested against the human tumor cell line MCF7 [Human Caucasian breast adenocarcinoma].

**Remarks**	**IC**_90 _(µg/mL)	**IC**_50 _(µg/mL)	***Sample Code***
61.9% at 100ppm	131.7	81.9	*2d*
8.8% at 100ppm	--------	--------	*3a*
50.2% at 100ppm	--------	--------	*3b*
61.1% at 100ppm	131.8	82.9	*3d*
33.7% at 100ppm	--------	--------	*4a*
58.6% at 100ppm	132.1	81.9	*4b*
10.7% at 100ppm	--------	--------	*5a*
20.9% at 100ppm	--------	--------	*5b*
42.9% at 100ppm	--------	--------	*6a*
4.4% at 100ppm	--------	--------	*6b*
9.8% at 100ppm	--------	--------	*7c*
60.7% at 100ppm	132.2	85.0	*7d*
55.4% at 100ppm	143.1	90.5	*8d*
0% at 100ppm	--------	--------	*9c*
56.5% at 100ppm	--------	--------	*9d*
44.7% at 100ppm	--------	--------	*10c*
44.5% at 100ppm	--------	--------	*10d*
2.9% at 100ppm	--------	--------	*11c*
52.9% at 100ppm	--------	--------	*11d*
31.2% at 100ppm	--------	--------	*12c*
57.5% at 100ppm	137.1	87.4	*12d*
3% at 100ppm	--------	--------	*DMSO*
*0 %*	*--------*	*--------*	*Negative control*

IC_50_: Lethal concentration of the sample which causes the death of 50% of cells in 48 h.

IC_90_: Lethal concentration of the sample which causes the death of 90% of cells in 48 h.

*Docking analysis*

Compounds 2d, 3a, 3b, 3d, 4a, 4b, 5a, 5b, 6a, 6b, 7c, 7d, 8d, 9c, 9d, 10c, 10d, 11c, 11d, 12c, 12d were used for docking study. All the calculations were performed using "Internal coordinate Mechanics" (Molsoft ICM 3.5-0a). Molecular modeling docking studies is performed and ICM score values (49-51) combined with hydrogen bonds formed with the surrounding amino acid residues help to predict the correct binding geometry for each binder at the active site. The molecular docking was performed into the hydrophobic site of EGFR with the aim to predict antitumor activity of compounds of the study (2d, 3a, 3b, 3d, 4a, 4b, 5a, 5b, 6a, 6b, 7c, 7d, 8d, 9c, 9d, 10c, 10d, 11c, 11d, 12c, 12d) against A549, HePG2 and MCF7 cell lines.

As shown in Table 5, Erlotinib (ligand) reveals ICM score of -90.54 and forms 3 H bonds with Met769, Cys773 and Gln767 (Figure 3), the target compounds elicited binding affinities (ICM scores range from -40.86 to -73.01). Compounds 10d, 12d, 8d, 11d, 9d showed activity probably due to their high ICM scores which ranged from -62.33 to -73.01 however compounds 4a, 6b, 7c, 5a are biologically inactive; they have low ICM scores of ranges from -40.86 to -50.44. 

**Table 5:** Docking of compounds on EGFR

**Length of H-bond Å**	**Amino acid residues forming the hydrogen bonds**	**Atom of ligand involved**	**No. of H-bonds**	**ICM score (∆G)**	***Cpd No***
1.66	Lys721	m of M o1	1	-50.98	*2d*
2.65 1.97	Thr766 Met769	m of M n1 m of M n2	2	-54.01	*3a*
2.73	Ile 758	m of M o3	1	-54.34	*3b*
1.64	Gln958	m of M o2	1	-56.61	*3d*
2.62 2.11 2.67	Lys721 Met769 Thr766	m of M n3 m of M o1 m of M h5	3	-50.44	*4a*
2.65 1.35	Thr766 Met769	m of M o2 m of M o1	2	-58.07	*4b*
2.08 1.82 2.37	Asn784 Ile 785 Gly 959	m of M o2 m of M o2 m of M n2	3	-40.86	*5a*
2.27 1.96	Gly 786 Gln 788	m of M n2 m of M o2	2	-53.34	*5b*
1.59 2.31 2.40 1.59 2.29 2.61	Lys721 Lys721 Glu738 Asp831 Asp831 Asp831	m of M n4 m of M n5 m of M h13 m of M h11 m of M h11 m of M h12	6	-50.45	*6a*
2.72 2.33 2.32 1.07 1.22 2.28	Asp 783 Gln958 Lys 782 Lys 782 Lys 782 Asp 783	m of M n3 m of M n2 m of M h12 m of M h13 m of M h14 m of M h12	6	-49.58	*6b*
2.32 1.94 2.62 2.50	Gln 677 Arg 752 Arg 807 Arg 807	m of M o1 m of M n3 m of M n2 m of M n2	4	-42.33	*7c*
2.39 1.46 2.19	Lys 782 Asp 783 Asp 783	m of M h16 m of M h15 m of M h16	3	-56.93	*7d*
2.78 2.05	Thr766 Met769	m of M o2 m of M o1	2	-66.51	*8d*
1.98	Met769	m of M o1	1	-59.05	*9c*
1.55	Asp 783	m of M h15	1	-62.33	*9d*
1.81	Asp 783	m of M h10	1	-54.63	*10c*
2.18 2.80 2.38	Lys721 Asp831 Asp831	m of M o1 m of M h15 m of M h16	3	-73.01	*10d*
1.53	Gln958	m of M o1	1	-50.55	*11c*
1.34 1.59	Gly 786 Glu961	m of M o2 m of M h15	2	-66.26	*11d*
2.46	Gln958	m of M n5	1	-56.70	*12c*
2.13 1.35	Gly786 Glu961	m of M n2 m of M h9	2	-68.71	*12d*
*1.90* *1.75* *2.01*	*Met769* *Cys773* *Gln767*	*m of M n3* *m of M o4* *m of M h7*	*3*	*-90.54*	*Erlotinib*

*Conclusion*

**Figure 4:**
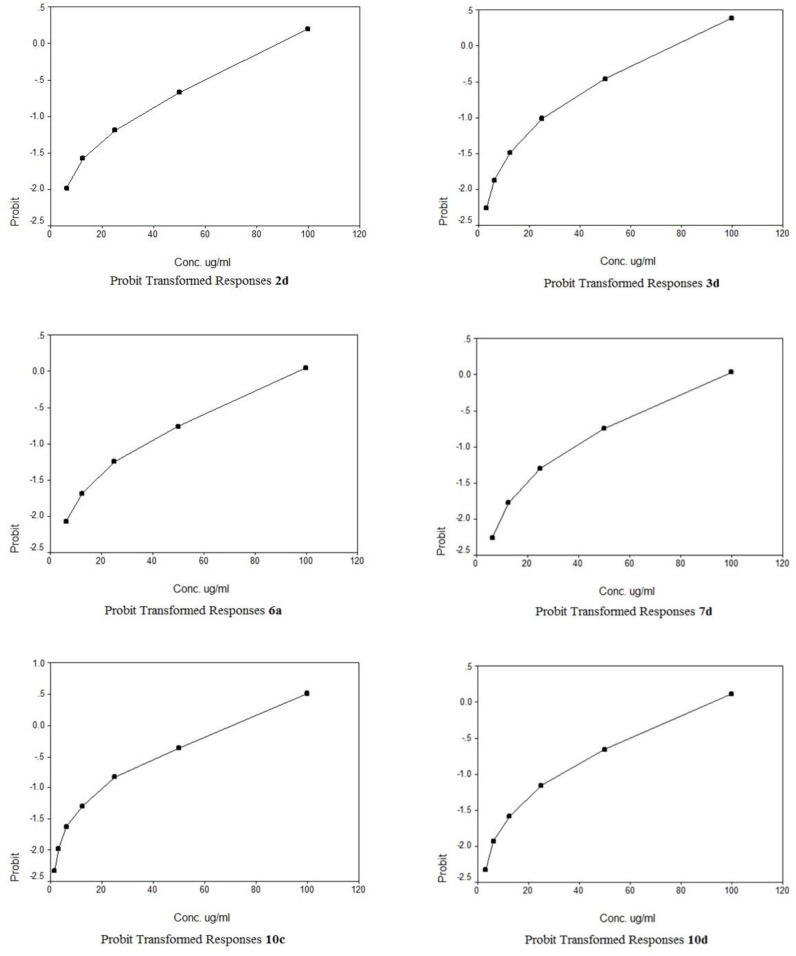
Probit Transformed Responses of some compounds against the human tumor cell line A549 [Lung carcinoma cell line

**Figure 5:**
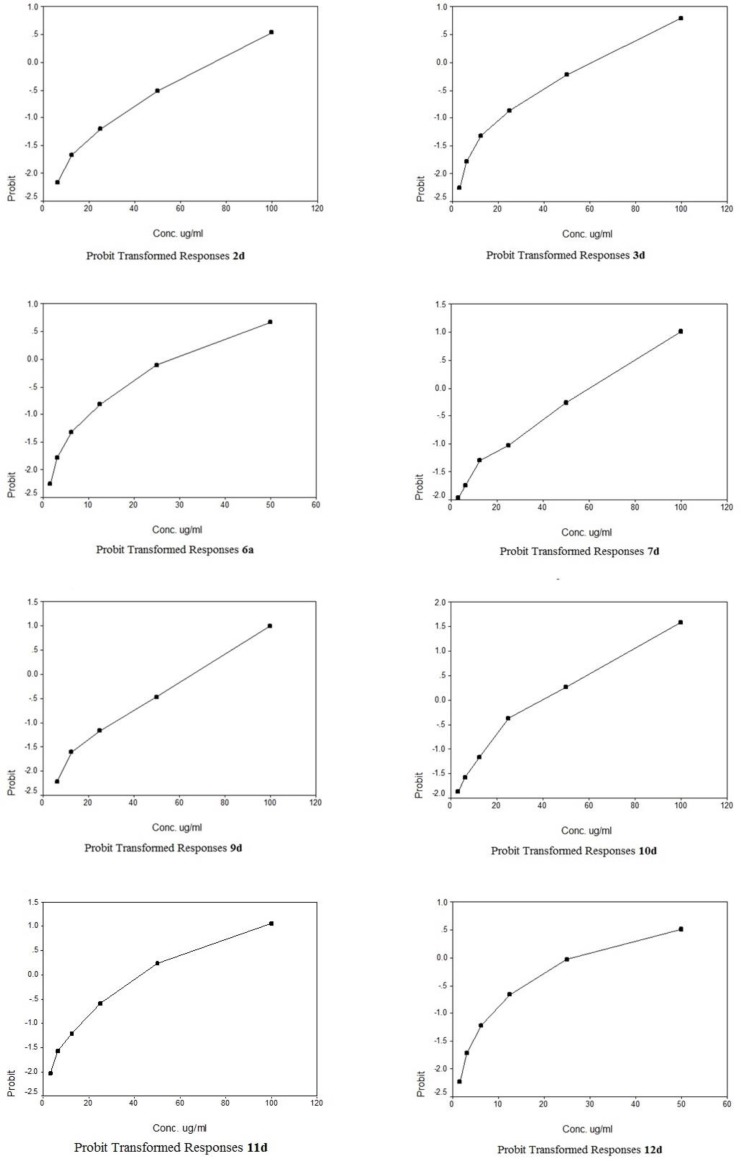
Probit Transformed Responses of some compounds against the human tumor cell line HePG2 [Human hepatocellular carcinoma cell line

**Figure 6:**
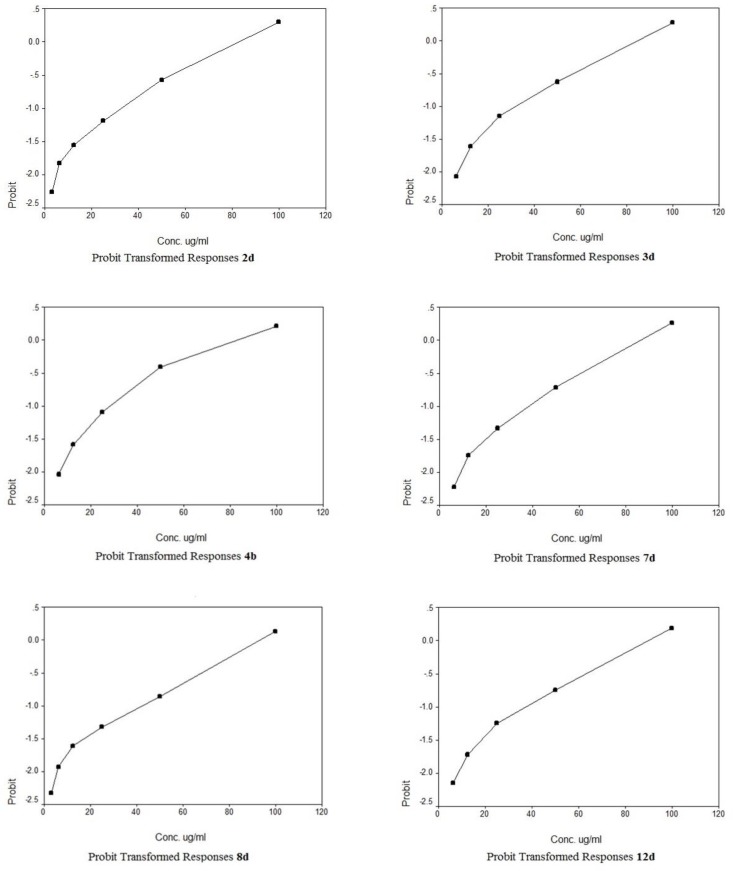
Probit Transformed Responses of some compounds against the human tumor cell line MCF7 [Human Caucasian breast adenocarcinoma

**Figure 7:**
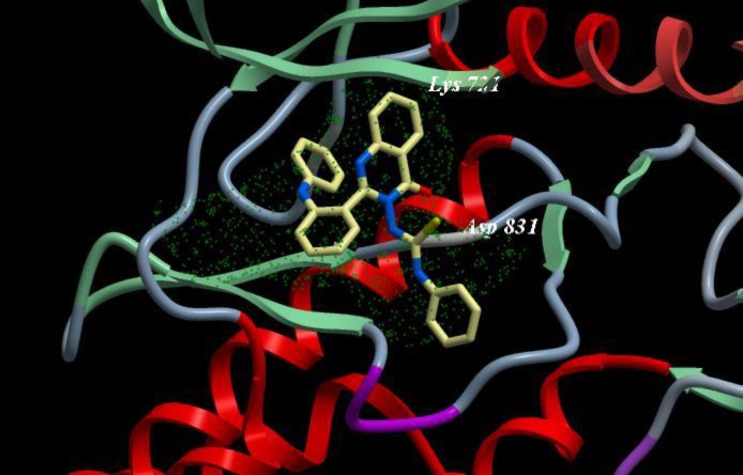
Binding mode of compound 10d with EGFR kinase. For clarity, only interacting residues are displayed. Ligand is represented as balls and sticks models and the green dots show the binding sites of EGFR

**Figure 8:**
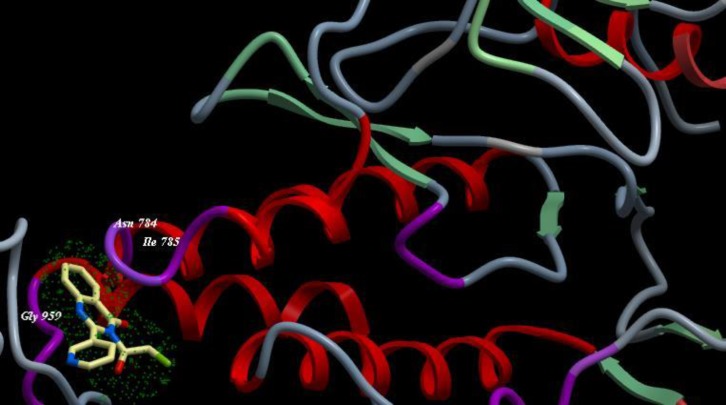
Binding mode of compound 5a with EGFR kinase. For clarity, only interacting residues are displayed. Ligand is represented as balls and sticks models and the green dots show the binding sites of EGFR

A novel series of some new quinazolin derivatives were synthesized and evaluated as antitumor agents against human carcinoma cell lines (HePG2– MCF7– A549). The antitumor activity results exhibited that, compounds 2d, 3d, 6a, 7d, 10c, 10d showed signiﬁcant and selective inhibition for A549 (Table 2) (Figure 4). On the other hand, compounds 2d, 3d, 6a, 7d, 9d, 10d, 11d, 12d showed signiﬁcant and selective inhibition for HePG2 (Table 3) (Figure 5). Compounds 2d, 3d, 4b, 7d, 8d, 12d showed signiﬁcant inhibition for MCF7 (Table 4) (Figure 6) comparing to the used reference drug Doxorubicin. Docking result shows that compound 10d have high ICM score -73.01 forms 3 H bonds with Lys721 and Asp831 (Figure 7). However, compound 5a has low ICM scores -40.86 forms 3 H bonds with Asn784, Ile 785 and Gly 959 (Figure 8).

*Structure-activity relationship*

The activity of the tested compounds could be correlated to structure variation and modiﬁcations. By investigating the variation in the selectivity of the tested compounds over the three cell lines, it was revealed that: (1) the activity of the designed compounds is dependent upon the substituent at the R positions. The obtained screening results showed that, nearly all of the compounds containing *N*-phenyl aniline showed signiﬁcant inhibition for the tested three cell lines (2). Cyclization of compound 2d afforded compound 3d (44) with the increase in activity against A549 with IC_50_ values 86.1 and 77.6 µg/mL, respectively, and for HePG2 with IC_50_ values 75.8 and 65.1 µg /mL, respectively, while result in a little decrease in activity against MCF7 with IC_50_ values 81.9 and 82.9 µg /mL, respectively (Table 2, 3, 4) (3). Compounds which have-CSNHPh group were found to be more active in the biological activities discussed in this paper than compounds which have –H. These results suggest that electron withdrawing hydrophilic substitutes (*e.g*.,-CSNHPh) are more desirable for achieving the desired activity. Also Certain isothiocyanates have also been shown to bind to the mutated p53 proteins found in many types of tumors, causing an increase in the rate of cell death (4). Compounds which have CO_2_CH_2_Cl yielded the least active series of compounds in this study. Which suggests that electron withdrawing groups with lipophilic characteristics like–Cl may not be an ideal substitution to get the good activity of the designed compounds.
